# Stable Isotopic Tracer Phospholipidomics Reveals Contributions of Key Phospholipid Biosynthetic Pathways to Low Hepatocyte Phosphatidylcholine to Phosphatidylethanolamine Ratio Induced by Free Fatty Acids

**DOI:** 10.3390/metabo11030188

**Published:** 2021-03-22

**Authors:** Kang-Yu Peng, Christopher K Barlow, Helene Kammoun, Natalie A Mellett, Jacquelyn M Weir, Andrew J Murphy, Mark A Febbraio, Peter J Meikle

**Affiliations:** 1 Metabolomics Laboratory, Baker Heart and Diabetes Institute, Melbourne, VIC 3004, Australia; Kang-Yu.Peng@hri.org.au (K.-Y.P.); chris.barlow@monash.edu (C.K.B.); Natalie.Mellett@baker.edu.au (N.A.M.); jacquio77@gmail.com (J.M.W.); 2 Department of Biochemistry and Molecular Biology, University of Melbourne, Parkville, VIC 3010, Australia; 3 Proteomics and Metabolomics Facility and the Department of Biochemistry and Molecular Biology, Biomedicine Discovery Institute, Monash University, Clayton, VIC 3800, Australia; 4 Hematopoiesis & Leukocyte Biology Laboratory, Baker Heart and Diabetes Institute, Melbourne, VIC 3004, Australia; helene.kammoun@mcri.edu.au (H.K.); Andrew.Murphy@baker.edu.au (A.J.M.); 5 Cellular & Molecular Metabolism Laboratory, Drug Discovery Biology, Monash Institute of Pharmaceutical Sciences, Monash University, Melbourne, VIC 3052, Australia; mark.febbraio@monash.edu; 6 Baker Department of Cardiometabolic Health, University of Melbourne, Parkville, VIC 3010, Australia

**Keywords:** lipidomics, free fatty acid, steatosis, flux analysis, CDP-choline pathway, CDP-ethanolamine pathway, phosphatidylethanolamine *N-*methyl transferase (PEMT) pathway

## Abstract

There is a strong association between hepatocyte phospholipid homeostasis and non-alcoholic fatty liver disease (NAFLD). The phosphatidylcholine to phosphatidylethanolamine ratio (PC/PE) often draws special attention as genetic and dietary disruptions to this ratio can provoke steatohepatitis and other signs of NAFLD. Here we demonstrated that excessive free fatty acid (1:2 mixture of palmitic and oleic acid) alone was able to significantly lower the phosphatidylcholine to phosphatidylethanolamine ratio, along with substantial alterations to phospholipid composition in rat hepatocytes. This involved both a decrease in hepatocyte phosphatidylcholine (less prominent) and an increase in phosphatidylethanolamine, with the latter contributing more to the lowered ratio. Stable isotopic tracer phospholipidomic analysis revealed several previously unidentified changes that were triggered by excessive free fatty acid. Importantly, the enhanced cytidine diphosphate (CDP)-ethanolamine pathway activity appeared to be driven by the increased supply of preferred fatty acid substrates. By contrast, the phosphatidylethanolamine *N*-methyl transferase (PEMT) pathway was restricted by low endogenous methionine and consequently low *S*-adenosylmethionine, which resulted in a concomitant decrease in phosphatidylcholine and accumulation of phosphatidylethanolamine. Overall, our study identified several previously unreported links in the relationship between hepatocyte free fatty acid overload, phospholipid homeostasis, and the development of NAFLD.

## 1. Introduction

Phospholipid plays a pivotal role in regulating physiological functions and maintaining cellular membrane structures, with phosphatidylcholine (PC) and phosphatidylethanolamine (PE) being the most abundant hepatocellular phospholipids [1,2,3]. A number of genetic knockout (e.g., *pemt* KO) and dietary (e.g., choline- and methionine-deficient diet) animal models indicated that phospholipids might be involved in the pathogenesis of non-alcoholic fatty liver disease (NAFLD) by altering the ratio of PC to PE, which subsequently leads to NAFLD [4,5,6].

Genetic polymorphisms are not the only possible trigger for the altered hepatic phosphatidylcholine to phosphatidylethanolamine ratio (PC/PE). A decreased hepatic PC/PE has been observed in a NAFLD patient study and high-fat diet fed mice [7]. In contrast to the low PC/PE observed in the abovementioned genetic knockout and choline- and methionine-deficient animal models, these reports appear to be more relevant to the pathogenesis of human NAFLD, which commonly involves obesity resulting from high caloric diets. Furthermore, an aberrant membrane PC/PE could also lead to NAFLD through ER stress [8]. Earlier reports indicated that ER stress could also be provoked by high levels of saturated fatty acid or low levels of polyunsaturated species, whereas monounsaturated fatty acids appeared to counter ER stress, inflammatory responses, and attenuate insulin signals induced by saturated free fatty acid [8,9]. Several radioisotope tracer studies also suggested that in vitro administration of free fatty acids (FFA) could alter the activities of specific hepatocyte phospholipid biosynthetic pathways, with different FFA species exerting dissimilar effects [10,11].

Stable isotopic tracers in combination with mass spectrometry and metabolomics (also known as metabolic flux analysis) are powerful tools for measuring the activities of biosynthetic pathways of interest [12,13]. By applying selected labelled precursor of the pathway of interest, flux analysis can be highly specific for tracing a biosynthetic pathway. It is also capable of detecting early changes in pathway activity before this altered pathway activity eventually leads to a difference in the metabolite pool, as newly synthesized “heavy” metabolite molecules can be detected and analyzed separately from the existing ones. Deuterated tracers have been widely applied to study PC and PE biosynthetic pathways. Some of the most important findings regarding fatty acyl preferences of different phospholipid biosynthetic pathways were made possible by the use of various deuterated tracers [14,15,16,17]. This approach has also been used to gauge changes to PC pathway activities under physiological conditions [16,18].

In this study, we observed a previously unreported decrease in rat hepatocyte PC/PE triggered by 1:2 mixture of palmitic (16:0) and oleic (18:1) acid, two of the most abundant intrahepatic FFA species found in NAFLD and a widely used agent to trigger in vitro steatosis [19,20,21]. Stable isotopic tracers and modulation of specific phospholipid biosynthetic pathways were also applied to further elucidate the underlying mechanisms behind the effects of excessive FFAs on hepatocyte phospholipidome.

## 2. Results

### 2.1. Effects of FFAs on Hepatocyte PC/PE

In an attempt to determine which FFAs and ratios of FFAs would provide a phospholipidomic phenotype similar to the one seen in NAFLD, we stimulated rat hepatocytes in differing FFA treatments. Indeed, several earlier studies have indicated that specific FFA species could differently affect phospholipid biosynthesis and thus alter phospholipid contents and composition [10,11,22,23]. To achieve this, we first examined the respective effects of palmitic acid (16:0), oleic acid (18:1), and a 1:2 mixture of the two FFAs (MFFA) on the PC and PE contents of hepatocytes. Palmitic acid (2 mM) significantly increased, while oleic acid (1.33 mM) appeared to decrease the total PC content of hepatocytes (Supplementary Figure S1A). Total PE, by contrast, only appeared to increase when treated with the mixture of palmitic and oleic acid (MFFA group, Supplementary Figure 1B). When compared to the control (Ctrl) group, 2 mM of MFFA decreased PC/PE by 24.7%, while 2 mM of palmitic acid increased the same ratio by 82.8% (Supplementary Figure S1C).

### 2.2. Concentration and Kinetic Effects of MFFA on Hepatocyte PC/PE

**Figure 1:**
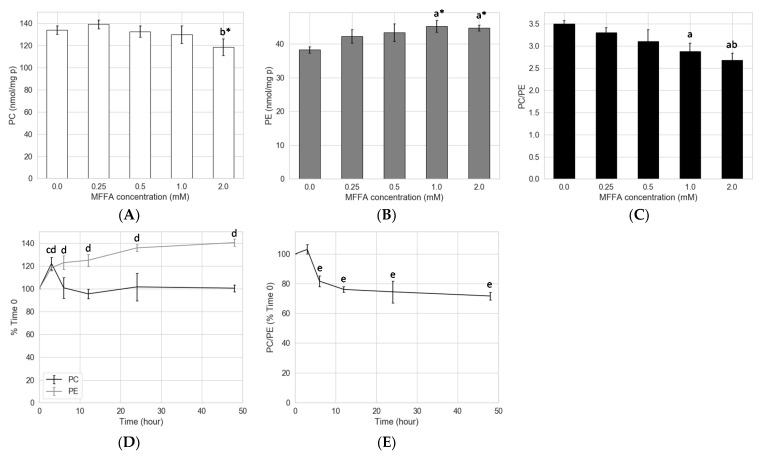
Effects of MFFA on hepatocyte phospholipid and PC/PE. MFFA treatments with different doses and durations were tested. Isolated rat hepatocytes were treated with 0–2 mM of MFFA for 24 h (dose effects, panel **A**–**C**), and 2 mM of MFFA for 0–48 h (time course effects, panel **D**,**E**). PC and PE contents (nmol/mg protein) in the cellular extracts were determined by LC-MS lipidomic analysis, and PC/PE was calculated accordingly. Data shown as mean ± sem. For dose effect experiments, a Kruskal–Wallis test was first performed followed by Mann–Whitney U tests as the *post hoc* analysis; for time effect experiments, Mann–Whitney U tests between control and MFFA groups at matched time points were performed; a: *p* < 0.05 vs. 0.0 mM group; b: *p* < 0.05 vs. 0.25 mM group; c: *p* < 0.05 vs. control group for PC at the labelled time point; d: *p* < 0.05 vs. control group for PE at the labelled time point; e: *p* < 0.05 vs. control group for PC/PE at the labelled time point. * indicates the comparison is only significant for *post hoc* tests, but not the Kruskal–Wallis test (*n* = 4).

Because the 1:2 mixture of palmitic and oleic acid (MFFA) was more pathologically relevant to NAFLD, we carried out additional experiments to test its effects on PC, PE, and the ratio of the two. Treatment with MFFA for 24 h mildly decreased hepatocyte total PC, with a significant difference only observed between the 0.25 and 2.0 mM groups (Figure 1A). An increase in total PE was observed in hepatocytes treated with 1.0 and 2.0 mM of MFFA (Figure 1B). Together these changes resulted in a dose-dependent decrease in PC/PE, which was significant when the MFFA was supplemented at 1.0 and 2.0 mM compared to 0.0 mM group (Figure 1C). To explore the kinetics of MFFA on hepatocyte phospholipid contents, we performed a time course experiment. MFFA initially triggered an increase in hepatocyte PC at 3-h post treatment, which returned to the baseline level within 6 h and stayed for the remainder of the experiment (i.e., 48 h) (Figure 1D). Conversely, hepatocyte PE content displayed a dramatic increase over the first 3 h, tapering off to steadily increase over the remaining 48 h (Figure 1D). This resulted in a significant lowering of the PC/PE from 6 to 48 h (Figure 1E).

**Figure 2:**
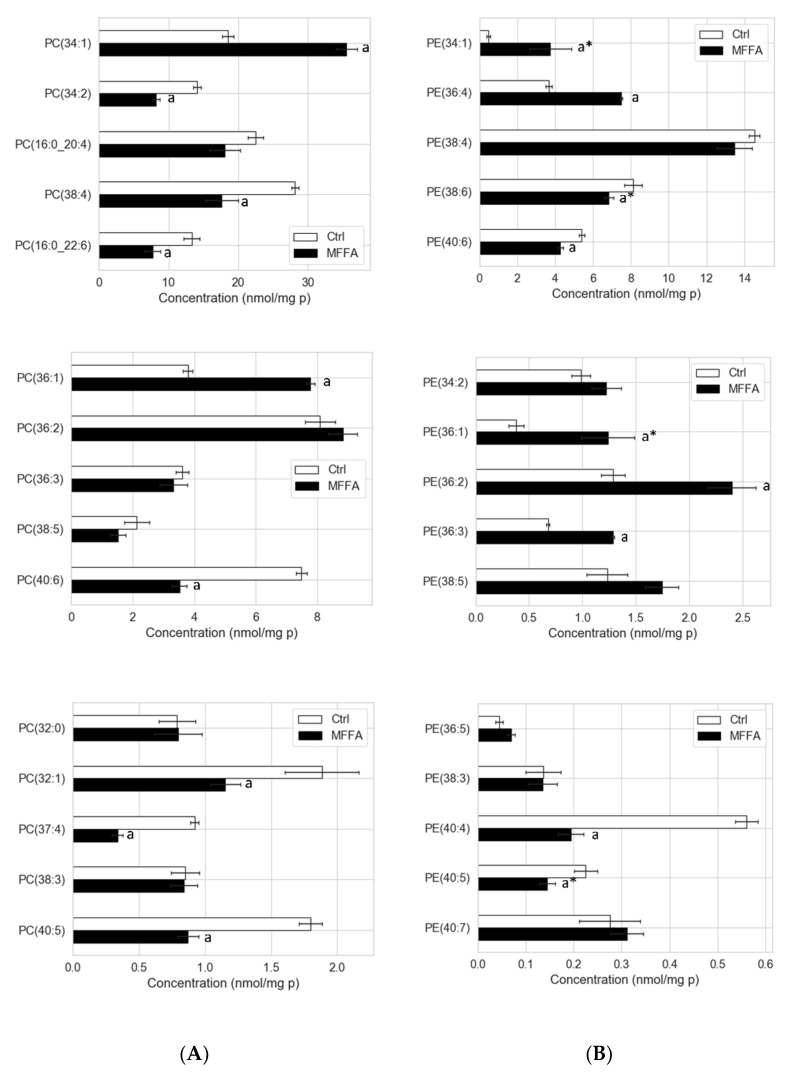
Dose effects of MFFA on the selected PC and PE species. Isolated rat hepatocytes were treated with 0 or 2 mM of MFFA for 24 h. The top 15 PC and PE species in concentrations (nmol/mg protein) were determined by LC-MS lipidomic analysis and shown here (**A**,**B**). Lipid species in each lipid class are separated into three panels based on their abundances. Data shown as mean ± sem; a: *p* < 0.05 vs. Ctrl (0.0 mM) group as determined by Mann–Whitney U test; * indicates the comparison is only significant for the *post hoc* test, but not the Kruskal–Wallis test (*n* = 4).

As expected, many of the PC and PE species consisting of palmitoyl and oleoyl chains e.g., PC(34:1), PC(36:1), PE(34:1), PE(36:1), PE(36:2), PE(36:3), PE(36:4) increased from approximately 50% to several fold of control in response to MFFA treatment (Figure 2A,B). A notable discrepancy found between PC and PE profiles was that polyunsaturated species were depleted more from the PC pool than the PE pool upon MFFA treatment. This was apparent when we compared PC and PE species with matched fatty acyl composition, such as PC(38:4) vs. PE(38:4), PC(16:0_22:6) vs. PE(38:6), PC(40:5) vs. PE(40:5), and PC(40:6) vs. PE(40:6) (Figure 2A,B). Also, the increment in PE(36:4) triggered by excessive MFFA was not seen in corresponding PC(16:0_20:4) species. As a result, when treated with MFFA, a substantial increase was only seen in total PE but not total PC, which in fact appeared to decrease slightly despite the increase in several palmitoyl and oleoyl species boosted by the MFFA treatment.

The abovementioned changes to hepatocyte phospholipid profile and PC/PE upon MFFA treatment were less likely to be driven by the secretion of PC and PE, as the secretion pattern indicated that MFFA-treated hepatocytes actually secreted more PE and less PC over time (Supplementary Figure S2 and Supplementary Table S1).

### 2.3. Stable Isotope Tracer Analysis of Hepatocyte Phospholipid Turnover

**Figure 3:**
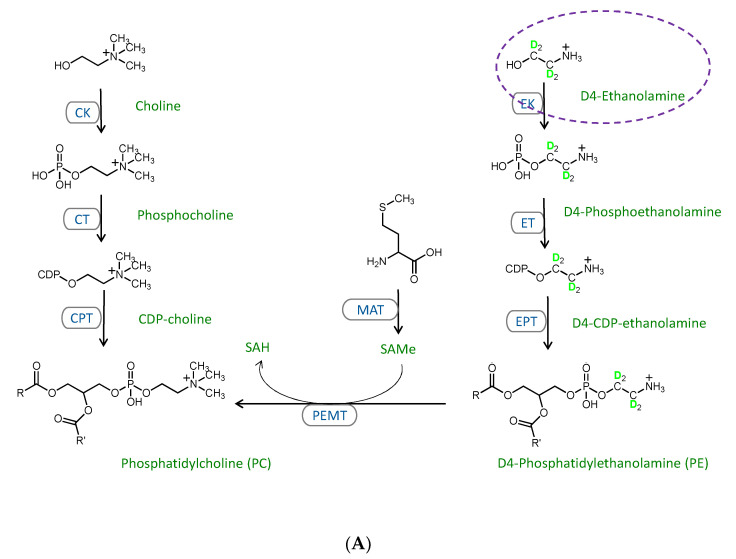

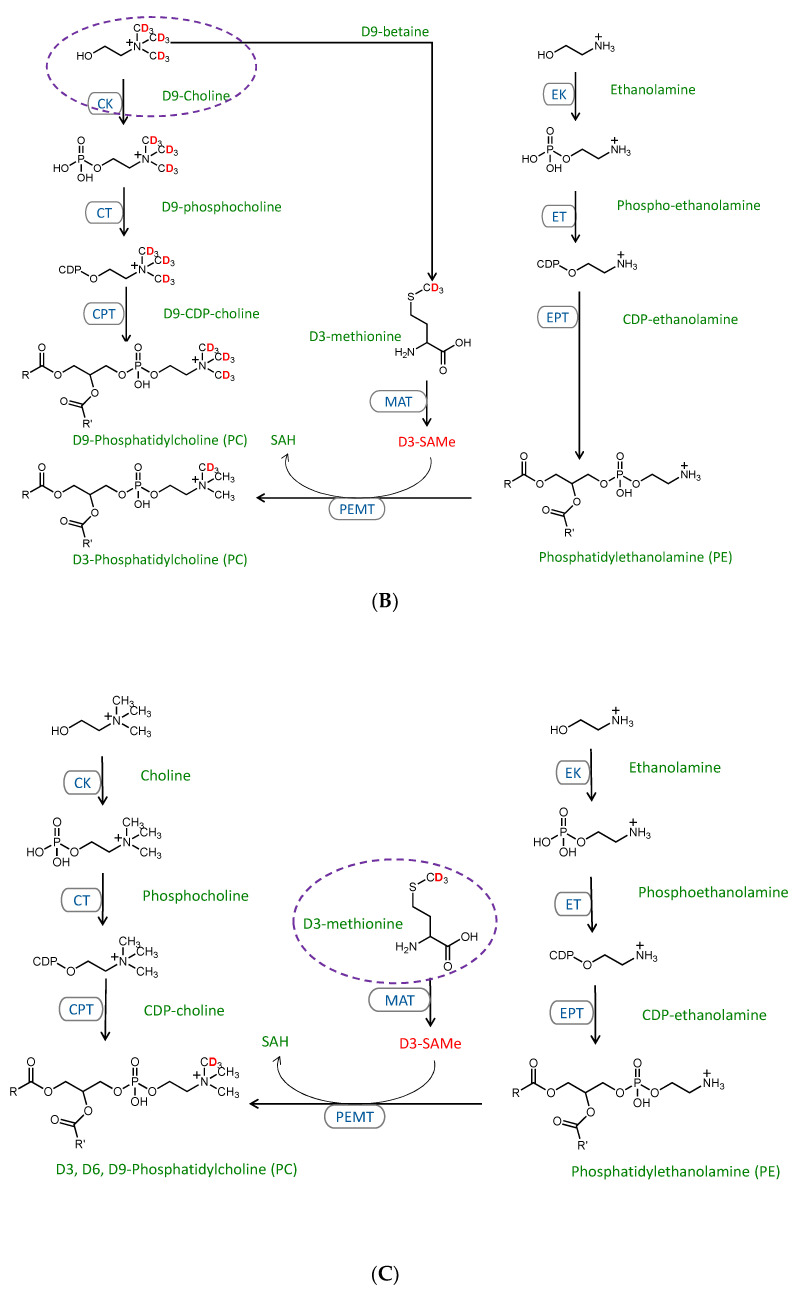
Schematic representations of traced phospholipid biosynthetic pathways in this study. Selected PC and PE biosynthetic pathways relevant to this study were obtained from the literature and the KEGG Pathway Database (https://www.kegg.jp/kegg/pathway.html; 15 January 2021). The deuterated tracers (D4-ethanolamine, D3-methionine, and D9-choline) used to substitute their hydrogenated counterparts in cell culture media are circled and the pathways they participate in are detailed. D4-ethanolamine traces the CDP-ethanolamine pathway (**A**); D9-choline can be used to trace the CDP-choline pathway (**B**). Notably, the supplementation of D9-choline can also give rise to D3-methionine, via betaine aldehyde and betaine [13,14,16], as shown in panel (**B)**. D3-methionine traces the PEMT pathway and can produce D3, D6, and D9 PC and LPC species (**C**). EK: ethanolamine kinase; ET: CTP:phosphoethanolamine cytidylyltransferase; EPT: CDP-ethanolamine:1,2-diacylglycerol ethanolaminephosphotransferase; PEMT: phosphatidylethanolamine N-methyltransferase; CK: choline kinase; CT: CTP:phosphocholine cytidylyltransferase; CPT: CDP-choline:1,2-diacylglycerol cholinephosphotransferase; SAMe: S-adenosyl-L-methionine; MAT: methionine adenosyltransferase.

While the phospholipid profiles suggested the involvement of phospholipid biosynthetic pathways in lowering the PC/PE and the disruption of the phospholipidome triggered by excessive MFFA, it failed to provide information regarding the relative contributions of these pathways. To overcome these limitations, we exploited flux analysis, a highly specific and sensitive measure that enabled us to separately examine the contribution of each phospholipid biosynthetic pathway and compare the newly synthesized phospholipid pool to the total phospholipid pool. D4-ethanolamine (1,1,2,2-D4), D9-choline (trimethyl-D9), and D3-methionine (methyl-D3) have been shown to label the cytidine diphosphate (CDP)-ethanolamine, CDP-choline, and PEMT pathways, as detailed in Figure 3 [14,15,16,17,18]. In vitro tracer experiments utilizing these stable isotopic precursors were performed by substituting culture media ethanolamine, choline, and methionine with their D4, D9, and D3 deuterated counterparts in the presence or absence of 2 mM MFFA for 6 h. D4 labelled (+4 *m*/*z*) PE and lysophosphatidylethanolamine (LPE) species were detected in D4-ethanolamine experiments, whereas D3-, D6-, and D9-labelled (+3, +6 and +9 *m*/*z*) PC, lysophosphatidylcholine (LPC) and sphingomyelin species were detected in D9-choline and D3-methionine experiments.

**Figure 4:**
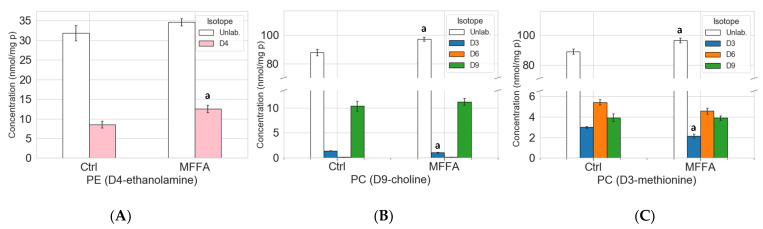
Isotopic labeling profiles for PE and PC in control and MFFA-treated hepatocytes. Isolated rat hepatocytes were treated without (Ctrl) or with 2 mM of MFFA in special DMEM media of which a nutrient has been replaced with its deuterated version (50 μM D4-ethanolamine, 30 μM D9-choline or 200 μM D3-methionine), serving as a flux tracer for phospholipid biosynthetic pathways, for 6 h. The cells were then harvested and underwent lipid extraction, followed by lipidomic analysis. (**A**) shows the concentrations of unlabeled (unlab.) and D4-labelled (D4) PE in the D4-ethanolamine labelling experiments (Data shown as mean ± sem; pink: D4-labelled PE; a: *p* < 0.05 vs. corresponding D4-labelled PE in Ctrl group, Mann-Whitney U test, *n* = 4). (**B**) and (**C**) show the concentrations of the unlabeled (unlab.) and D3, D6, and D9-labelled (D3, D6, and D9) PC in D9-choline and D3-methionine labelling experiments, respectively. (Data shown as mean ± sem; discrete *Y*-axis; blue: D3-labeled PC; orange: D6-labeled PC; green: D9-labeled PC; a: *p* < 0.05 vs. corresponding unlabeled/labeled PC in Ctrl group; Mann–Whitney U test; *n* = 3 for D9-choline and D3-methionine experiments).

MFFA significantly increased intracellular D4-PE, while the unlabeled PE did not change significantly (Figure 4A and Supplementary Table S2). This result suggested that MFFA treatment led to the accumulation of newly synthesized PE molecules and a consequent increment of total hepatocyte PE, potentially via the CDP-ethanolamine pathway.

The D9-choline tracer not only generates D9 phospholipid species via the CDP-choline pathway, but also D3 and D6 species via the production of the methyl donor D3-*S*-adenosylmethionine which feeds into the PEMT pathway [15,18] (Figure 3B). Thus, D3- and D6-labeled PC species are indicative of the PEMT pathway activity in this experiment. Both unlabeled and D9-labeled PC increased in response to MFFA treatment (by 10.4% and 8.4%; the latter non-significant). D3-labeled PC, unlike the unlabeled and D9-labeled PC, decreased significantly (Figure 4B). This profile aligned well with the 6-h time point PC profile in the earlier kinetics experiments (Figure 1D and Supplementary Table S3). The data also indicated that PC breakdown and/or exportation was suppressed, at least at this particular time point, as unlabeled PC elevated in MFFA group vs. Ctrl group. The decline of D3-PC suggested that the PEMT pathway was less active when treated with excessive MFFA. We have also observed a decrease in unlabeled as well as D3- and D9- labeled LPC with MFFA treatment when compared to the control group, which indicated that MFFA may also have an impact on PC degradation (Supplementary Figure S3B and Supplementary Table S4).

D3-methionine effectively traced the PEMT pathway for PC production, as shown in Figure 4C and Supplementary Table S5. We first identified that there was a substantial amount of D3 and D6 phospholipid, which combined was more than the D9 phospholipid for all three lipid classes (Figure 4C, Supplementary Figure S3C and Supplementary Table S5). This observation suggested that endogenous methionine was a major source of *S*-adenosylmethionine, the methyl donor for PEMT and many other methyltransferases, as the exogenous source of this amino acid from culture media was completely D3-methionine [24]. Similar to the D9-choline tracer experiments, the LPC decreased in the presence of MFFA, with a significant decline seen in unlabeled and D3-labeled LPC (Supplementary Figure S3C). Also similar to the D9-choline experiment was the difference in PC levels between control and MFFA-treated groups, with unlabeled PC increasing significantly and D3-labelled PC decreasing significantly in the MFFA treatment group (Figure 4C). These results indicate that MFFA treatment can disrupt both the existing (unlabeled) and newly synthesized (labelled) phospholipid pools in hepatocytes.

### 2.4. Phospholipid Isotopic Enrichment Profiles

**Figure 5:**
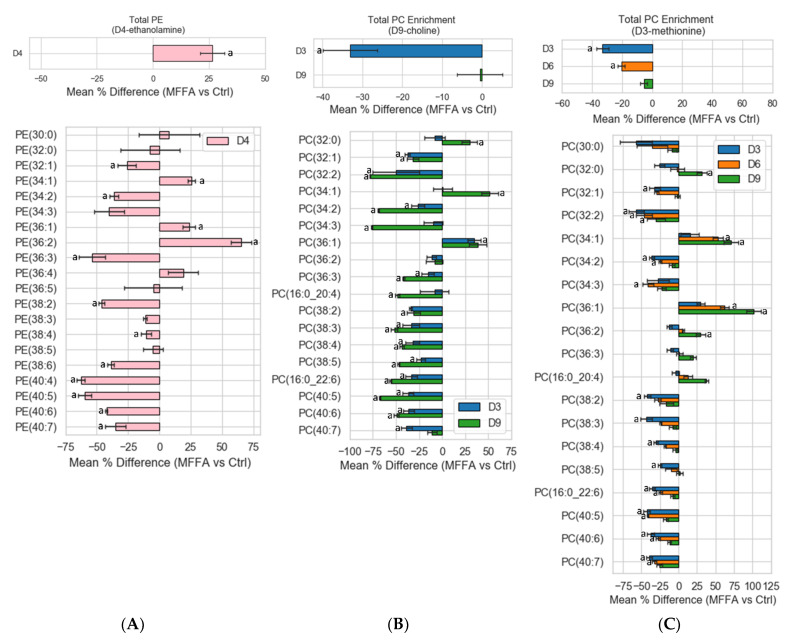
Differences in isotopic-labeled lipid enrichments between control and MFFA-treated hepatocytes. Isolated rat hepatocytes were treated without (Ctrl) or with 2 mM of MFFA in special DMEM media of which a nutrient has been replaced with its deuterated version (50 μM D4-ethanolamine, 30 μM D9-choline, or 200 μM D3-methionine), serving as a flux tracer for phospholipid biosynthetic pathways. The cells were then harvested after 6 h. Mean percentage differences in enrichment values of the deuterated PE for D4-ethanolamine (**A**) and deuterated PC for D9-choline (**B**) and D3-methionine (**C**) experiments are shown. Phospholipid species with very low abundances which made the calculation of enrichments and mean % differences impossible or unreasonable (0 as denominator of the ratio or differences by several hundred folds) were excluded (a: *p* < 0.05 Ctrl vs. MFFA enrichments, Mann–Whitney U test; *n* = 4 for D4-ethanolamine experiments; *n* = 3 for D9-choline and D3-methionine experiments).

While the phospholipid class concentrations and percentages of control data revealed some differences in specific phospholipid biosynthetic pathways between Ctrl and MFFA groups, one caveat is that simultaneous degradation and exportation could confound the interpretation of changes in phospholipid biosynthesis using the concentration data. However, it is not possible to monitor all the phospholipid degradation mechanisms and so to minimize the influence of degradation we converted the concentration data into enrichment, i.e., the ratio of labelled lipid to the sum of labeled and unlabeled lipid [16,18]. The mean % difference (MFFA vs. Ctrl) enrichment data of phospholipid labelling experiments are presented in Figure 5.

Similar to the concentration data for the D4-ethanolamine experiments, upon MFFA treatment the enrichment of total D4-PE increased significantly, which indicated an enhanced CDP-ethanolamine pathway (Figure 5A, top panel). Labelling enrichment data of the PE species provided further information regarding the effects of excessive MFFA. For D4-ethanolamine experiments, significantly more saturated and monounsaturated D4-PE species were detected, while many polyunsaturated species were diminished (Figure 5A, bottom panel).

For labelled PC enrichments in the D9-choline experiments, only D3-PC decreased significantly in response to MFFA, but not D9-PC. This indicated that the PEMT pathway was suppressed while the CDP-choline pathway was largely unaffected (Figure 5B, top panel). Furthermore, several distinctions could be seen between D3- and D9-labelled PC species, as they respectively represented biosynthesis via the PEMT and CDP-choline pathways. For instance, the enrichment of D3-PC(32:0) and D3-PC(34:1) barely changed while the synthesis of D9-PC(32:0) and D9-PC(34:1) increased most by excessive MFFA (Figure 5B, bottom panel). The reduction in D3-polyunsaturated PC biosynthesis in reaction to MFFA also generally was not as dramatic as the D9-polyunsaturated PC species, with PC(40:7) being an exception (Figure 5C). These results largely fit the preferences for fatty acyl chain usage by these pathways.

One of the more interesting observations was in the D3-methionine labelling studies, which specifically labelled PC via the PEMT pathway. As mentioned before, we detected abundant D3 and D6 phospholipid in addition to D9 phospholipid. Surprisingly, the labelled PC enrichment profile suggested that the number of labelled methyl groups constituting labelled PC inversely correlated with the suppression of PC production via the PEMT pathway i.e., the suppression of the PEMT pathway triggered by MFFA was most apparent among D3-PC, to a lesser extent for D6-PC, and were not observed for D9-PC (Figure 5C, top panel). This inverse relationship between the mean % differences enrichments of D3-, D6-, and D9-PC was significant (Kruskal–Wallis test). Enrichment data from D3-methionine labelling experiments can be viewed as a surrogate for the PEMT pathway activity. D3-PC species acted very similarly to their counterparts found in the D9-choline labelling experiments (Figure 5B,C), which confirmed that the PEMT pathway was less active in the presence of MFFA. Similar to PC lipid class data, the trend in D3-, D6-, and D9-PC were still observed at the lipid species level. Notably, this D3 < D6 < D9-PC enrichment trend was followed by almost all the PC species despite dramatic changes to their relative abundances (as indicated by mean % difference enrichments) in reaction to MFFA treatment (Figure 5C, bottom panel). Mean % difference enrichments between D3, D6, and D9 species listed below were significant (Kruskal–Wallis test): PC(32:0), PC(34:2), PC(36:1), PC(36:2), PC(36:3), PC(16:0/20:4), PC(38:3), PC(38:4), PC(16:0/22:6), and PC(40:6). Significant increase in the enrichments of labelled palmitoyl and oleoyl containing PC species as well as decrease in labelled polyunsaturated PC species reflected the utilization of administrated MFFA by PEMT (Figure 5C, bottom).

### 2.5. Effects of Altered Media Phospholipid Precursors on the Hepatocyte Lipidome and Cell Death

**Figure 6:**
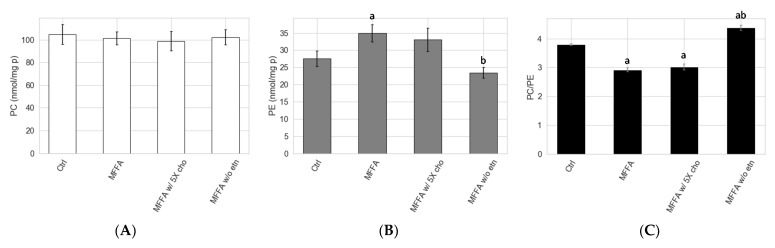
Impacts of altered supplies of key nutrients involved in phospholipid biosynthesis on MFFA-treated hepatocyte phospholipid and PC/PE. Isolated rat hepatocytes were treated without (Ctrl) or with 2 mM of MFFA in special DMEM media of which choline was increased to five times of the standard doses (i.e., 150 μM, MFFA w/5X cho), or had ethanolamine removed completely (MFFA w/o etn) for 24 h before being harvested and lipid extraction. The cellular PC and PE profiles were subsequently determined by LC-MS lipidomic analysis (**A**,**B**), and PC/PE was calculated accordingly (**C**). Data shown as mean ± sem; a: *p* < 0.05 vs. Ctrl group; b: *p* < 0.05 vs. MFFA group, both determined by Mann–Whitney U test (*n* = 5 for Ctrl, MFFA and MFFA w/o etn groups; *n* = 4 for MFFA w/5X cho group).

**Figure 7:**
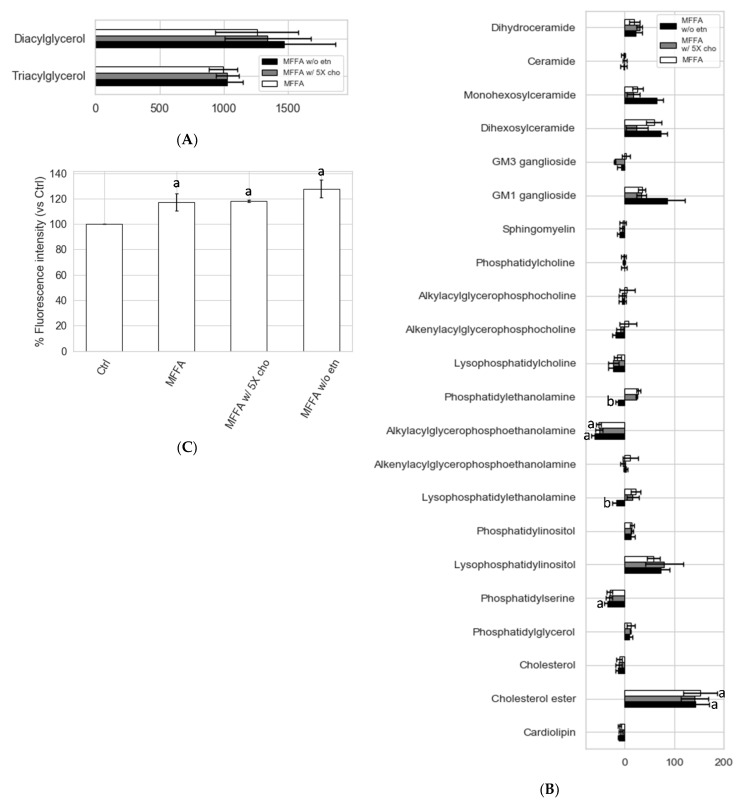
Impacts of altered supplies of key nutrients involved in phospholipid biosynthesis on steatotic hepatocyte lipid profile and cell death. Isolated rat hepatocytes were treated without (Ctrl) or with 2 mM of MFFA in special DMEM media of which choline has been increased to five times of the standard doses (i.e., 150 μM, MFFA w/5X cho), or had ethanolamine removed completely (MFFA w/o etn) for 24 h, before being harvested and LC-MS lipidomic analysis performed. Mean percentage differences (vs Ctrl) of the lipid classes under different treatment conditions are shown (**A**,**B**; data shown as mean % difference ± sem); a: *p* < 0.05 vs. Ctrl group, as determined by a Kruskal–Wallis test followed by Mann–Whitney U tests; *p*-values were Benjamini–Hochberg corrected; *n* = 5 for Ctrl, MFFA and MFFA w/o etn groups; *n* = 4 for MFFA w/5X cho group). For cell death determination, rat hepatocytes were treated with the abovementioned media for 48 h before being trypsinized and stained with propidium iodide dye and analyzed with a flow cytometer. Relative fluorescence intensities vs. Ctrl (as 100%) are shown as (**C**) (data shown as mean ± sem; a: *p* < 0.05 vs. Ctrl group; b: 0.05 < *p* < 0.075 vs. MFFA group, Mann–Whitney U test; *n* = 4 for Ctrl, MFFA and MFFA w/o etn groups; *n* = 2 for MFFA w/5X cho group).

The low PC/PE triggered by MFFA could be corrected by controlling the supply of precursors that directly contribute to the phospholipid biosynthetic pathways. We sought to reverse the MFFA-induced low PC/PE state in hepatocytes by: (a) adding 5 times of the standard choline concentration; and (b) removing media ethanolamine in DMEM media. The addition of choline exerted little effect on PC/PE (Figure 6). The removal of ethanolamine, by contrast, effectively reduced hepatocyte PE production and restored hepatocyte PE to a level similar to the Ctrl group (Figure 6B). PC/PE level consequently became higher than both MFFA and Ctrl groups (Figure 6C). The lipidomic profile revealed that the effects of media ethanolamine removal were very specific to PE and LPE (*p* = 0.073, MFFA vs. MFFA w/o etn) (Figure 7 and Supplementary Table S6). Other than these changes, the treatment had few additional effects on the lipidome of MFFA-treated hepatocytes. The use of ethanolamine-free media did not ameliorate steatosis, as hepatocyte di- and triacylglycerol levels were unaltered as compared to the MFFA group (Figure 7A). This nutritional approach also failed to reverse MFFA-induced cell death, as determined by propidium iodide staining (Figure 7C).

## 3. Discussion

### 3.1. Prolonged Treatment with Excessive MFFA Lowers Hepatocyte PC/PE

It has been reported that patients with type II diabetes and obesity tended to have elevated circulating FFA [25]. In addition, due to its proximity and direct portal vein connection, the liver is frequently exposed to a higher-than-circulation level of FFA from visceral adipose tissue and/or diet, which has been shown to contribute to hepatic insulin resistance and NAFLD [26,27]. With a widely used in vitro model that mimicked the exposure of liver to excessive saturated and monounsaturated free fatty acids that commonly occurred in NAFLD, we demonstrated here that MFFA was able to significantly lower hepatocyte PC/PE mainly by increasing cellular PE. MFFA may also lower cellular PC, although this effect appears to be less reproducible (Figure 1). The effect of MFFA was not shared by the sole use of either palmitic or oleic acid. Palmitic acid increased hepatocyte PC and oleic acid appeared to decrease hepatocyte PC when a relatively low dose (1.33 mM) was administered. Intriguingly, unlike MFFA, either alone did not change the total PE contents. (Supplementary Figure S1). A similar decline in PC/PE has been reported in at least one study looking at a human NAFLD cohort and mice fed a high fat diet [7]. We have also shown that the amount of secreted PC and PE were unlikely to be major contributors to the low cellular PC/PE, and in fact, prevented a more dramatic decline in this ratio (Supplementary Figure S2).

### 3.2. CDP-Ethanolamine and PEMT Pathways Contribute to the Altered PC/PE

Stable isotopic tracer experiments revealed several previously unidentified effects of MFFA on phospholipid metabolism. Notably, changes to the biosynthetic rates appeared to influence PE more than PC, which is possibly due to a smaller intracellular pool size of the former (approximately 1/3) in hepatocytes. As revealed in the stable isotopic tracer experiments, both the CDP-ethanolamine and PEMT pathways contributed to the increment of hepatocyte PE in response to MFFA (Figure 5). In addition, the diminished production of PC via the PEMT pathway (in some settings) explains the subtle but dose-dependent decrease in cellular PC, which contributes to the decrease in the PC/PE in steatotic hepatocytes (Figure 1A). The observation that removing ethanolamine from the media prevented these changes to the hepatocyte PC/PE induced by MFFA further supports the CDP-ethanolamine pathway as the major contributor to this effect (Figure 7B).

### 3.3. Mechanisms Contributing to the MFFA-Induced Disruptions to CDP-Ethanolamine and PEMT Pathways

Hepatic phospholipid biosynthetic pathways preferentially produce phospholipid species containing specific fatty acyl groups under physiological conditions [14,15,16,17], with the CDP-ethanolamine pathway predominantly producing PE species with mono- or di-unsaturated fatty acyl group on sn2 position, such as PE(16:0_18:1) and PE(18:0/18:2)/PE(18:1/18:1) [17]. Our tracer phospholipidomic results provide one of the first mechanistic links between the preference to fatty acid substrates by the CDP-ethanolamine pathway to substantial increase in hepatocyte PE triggered by MFFA.

CDP-choline and PEMT pathways likewise have a preference for FFA substrates. Our results indicate that these pathways also incorporate saturated and monounsaturated FFAs when there is a high availability, with more palmitoyl and oleoyl PC produced via the CDP-choline pathway, as these are the preferred substrates of this pathway. However, unlike the CDP-ethanolamine pathway, alterations to PC composition with palmitic and oleic acid do not seem to have an impact on the CDP-choline pathway activity (Figure 4B and Figure 5B). The slight drop of cellular PC with prolonged MFFA treatment is likely resulted from the decreased production of PC via the PEMT pathway (Figure 5 and Supplementary Tables S4 and S5). Based on the D3-methionine tracer enrichment data, the lack of endogenous methionine and subsequently *S*-adenosylmethionine, the methyl donor which serves as the substrate for PEMT, is the main contributor to MFFA-induced suppression to the PEMT pathway. The enzymatic activities of PEMT and methionine adenosyltransferase (enzyme that catalyzes the reaction for *S*-adenosylmethionine production, see Figure 3C) were likely unhindered by MFFA, as they still effectively used methionine from extracellular source (D3-methionine) and the enrichment level of total D9-PC did not differ significantly between Ctrl and MFFA groups (Figure 5C and Supplementary Table S5). With these assumptions and knowledge of significant differences in D3- and D6-PC enrichment (MFFA group being 67.2% and 79.7% of Ctrl group, respectively) in D3-methionine experiments, we estimate that endogenous methionine decreased by roughly 16.4–20.3% upon MFFA treatment. Low intrahepatic methionine or *S*-adenosylmethionine is closely related to alcoholic fatty liver disease and NAFLD as has been demonstrated in several strains of genetic knockout mice, and can be alleviated by supplementing the methyl donors [28,29,30]. There is a less clear link between hepatic levels of methionine and *S*-adenosylmethionine and high-fat-diet-induced steatosis. However, some studies do report a relatively small (non-significant) decrease in hepatic methionine or *S*-adenosylmethionine level triggered by a high fat diet [31,32]. Our in vitro study agreed with these in vivo findings. The production of PC via the PEMT pathway is only slightly influenced under steatosis, so long as there is a sufficient supply of methionine from the outer sources (e.g., diet), as PEMT enzymatic activity is likely unhindered. This low intracellular methionine state may nonetheless predispose hepatocytes to second insults (e.g., choline- and methionine-deficient diet) and result in more severe steatosis or inflammation, which complies with the classic “Two Hits” model which describes NAFLD pathogenesis [33]. Methionine is an essential amino acid which participates in protein metabolism [34]. Furthermore, methionine and/or *S*-adenosylmethionine are required by the hepatic methyltransferases, DNA-methylation, and the replenishment of glutathione, which all have been shown to involve in the pathogenesis of NAFLD [6,34,35,36]. It would be interesting to evaluate the involvements of these mechanisms in the attenuated intracellular methionine level induced by MFFA that could eventually suppress the PEMT pathway.

### 3.4. Hepatocytes Show Tolerance to Aberrant PC/PE Induced by MFFA

Removing media ethanolamine suppressed the production of PE and prevented the low PC/PE triggered by MFFA treatment (Figure 6). Ethanolamine is not a standard ingredient for most of the commonly used cell culture media, but is a nutrient that exists in plasma and therefore is circulated to the liver [10]. Our study demonstrates that the lack of ethanolamine can have an impact on the hepatocyte phospholipidome, especially to the PE and LPE species. Except for lowering hepatocyte PE and LPE, removing media ethanolamine had few effects on the lipidome of steatotic hepatocytes (Figure 7A,B and Supplementary Table S6). Removing media ethanolamine also failed to rescue cell death triggered by MFFA, a potential consequence of “leaky cell membrane” resulted from low plasma membrane PC/PE [3,4]. It is possible that a more dramatic change to the hepatocyte PC/PE than the extent that can be triggered solely by excessive MFFA is required to cause noticeable disruptions to intracellular lipid metabolism and cell viability. In addition, hepatocytes also appear to be able to self-regulate cellular phospholipid by changing the amount and composition of secreted phospholipid, which may prevent a further attenuation to the PC/PE (Supplementary Figure S2 and Supplementary Table S1). Similar scenarios have been reported in studies carried out on PEMT knockout and methionine adenosyltransferase 1A knockout mouse models which both had impaired PC biosynthesis via the PEMT pathway. These rodents often were asymptomatic and even had nearly normal hepatic PC/PE. However, lacking these genes made the rodents more susceptible to second insults (e.g., choline and/or methionine-deficient diet) [4,37]. As demonstrated in a PEMT knockout mice study, when these mice were fed a choline-deficient diet, as much as 50% decrease in hepatic PC/PE occurred, accompanied by prominent signs of NAFLD [4]. Although mechanisms behind MFFA-induced low PC/PE reported in our study are different from the above genetic knockout models, this “preconditioned” low PC/PE state may similarly predispose hepatocytes to further insults that eventually lead to steatohepatitis, as reported in the knockout mice models. Finally, the involvement of other factors such as the fatty acyl composition of the phospholipid cannot be ruled out, as MFFA treatment inevitably altered fatty acyl profiles of PC and PE, not just their ratio. Altered phospholipid fatty acyl composition (increased saturated and monounsaturated species; decreased polyunsaturated species) may have contributions in addition to PC/PE, through mechanisms such as ER stress [8,9]. Future studies could focus on identifying additional insults which synergistically cause signs of NAFLD and notable changes to hepatocyte lipidome and PC/PE with MFFA, and simultaneously monitor changes to the fatty acyl composition of phospholipidome to clarify their respective contributions.

## 4. Materials and Methods

### 4.1. Materials

A full list of materials and reagents used in this study can be found in the Supplementary Information.

### 4.2. Rat Primary Hepatocytes Isolation, Culture, and Fatty Acid Treatment

All animal experiments were approved by the Alfred Medical Research and Educational Precinct Ethic committee (E/1440/2014/B). A two-step hepatic collagenase perfusion method was used for rat hepatocyte isolation, following the protocol of Foretz, et al. [38] and is detailed in the Supplementary Information.

All cell experiments, unless otherwise stated, were carried out in DMEM containing 5000 U penicillin/streptomycin, 3.7 mg/mL sodium bicarbonate and 50 μM ethanolamine. The purpose of ethanolamine supplementation to the media was to provide adequate substrate for PE synthesis via hepatocyte CDP-ethanolamine pathway. This also made cell culture condition consistent with the D4-ethanolamine labelling experiments. As a reference, blood ethanolamine concentration taken from rat portal vein has been reported to be between 18.9 and 23.1 μM [10]. Furthermore, ethanolamine concentrations as high as 400–800 μM have been used in cell culture media (flux analysis) [13,14,16]. For flux analyses, a special DMEM was used so that media hydrogenated choline, methionine, or ethanolamine could be substituted with its deuterated counterpart. In short, the special DMEM was first supplemented with 4 μg/mL calcium D-pentothenic acid, 7 μg/mL inositol, 110 μg/mL sodium pyruvate, 5000 U penicillin/streptomycin, and 3.7 mg/mL sodium bicarbonate. Depending on the intended labelling, 30 μM D9-choline chloride, 200 μM D3-L-methionine, or 50 μM D4-ethanolamine was added into the special DMEM. Afterwards, the other two hydrogenated nutrients (other than the tracer) were supplemented.

FFA complexed to BSA used to induce steatosis was prepared prior to administration to hepatocytes culture, largely as described by Karaskov, et al. [39]. As a reference, circulating FFA concentration could reach 1.3 mM during prolonged fasting [25]. Therefore, it is likely for hepatocytes to be exposed to 1–2 mM of FFA especially due to their close proximity to visceral adipose tissue and the connection of portal vein to the liver [26,27]. Diabetes and obesity may further increase the FFA level the liver is exposed to [25,27]. Detailed steps for the preparation can be found in the Supplementary Information.

### 4.3. Sample Preparation and Lipid Extraction

To prepare cell lysates for lipid extraction, 1.2 × 10^6^ rat hepatocytes were first trypsinized and suspended in 200 μL Tris-NaCl containing 100 μM of the antioxidant butylhydroxytoluene prior to probe sonication (Misonix, amplitude 26, 30 s on ice). To determine sample protein concentrations, the BCA assay was performed following the manufacturer’s instruction. Sample concentrations were subsequently adjusted to 2 mg/mL for lipid extraction. Culture media were collected at selected time points. Afterwards, 400 μL of culture media was centrifuged (15,000× *g*, 10 min) to remove any remaining cell debris. The supernatants were then lyophilized and were ready for lipid extraction. Prior to lipid extraction, the samples were redissolved in 10 μL of water to maintain a 1:20 water:organic solvent ratio.

An established single phase lipid extraction method for biological samples using CHCl_3_:MeOH (2:1) was performed in this study, following an earlier report [40]. Briefly, 10 μL samples were taken and combined with 200 μL CHCl_3_:MeOH (2:1) and 10 μL internal standard mix, followed by a quick vortex. Samples were then mixed with a rotary mixer for 10 min, water bath sonicated for 30 min and then left to stand at room temperature for 20 min. Afterwards, samples were centrifuged (16,000× *g*, 10 min). The supernatants were taken, transferred to a 96-well plate and dried with a Speedy Vac System (Thermo Fisher Scientific, Waltham, MA, USA). Extracted lipids were reconstituted by adding 50 μL water saturated butanol and then 50 μL MeOH containing 10 mM ammonium formate, followed by centrifugation (3350× *g*, 5 min, room temperature). Supernatants containing the reconstituted lipids were transferred to glass vials with glass inserts for lipidomic analysis.

### 4.4. Lipidomic Analysis

Liquid chromatography tandem mass spectrometry (LC-MS/MS) was used for targeted lipidomic analysis, similar to our earlier studies [41,42]. Briefly, an Agilent 1200 series HPLC system with a Poroshell 120 EC-C18 column (2.1 × 100 mm 2.7 micron, Agilent Technologies, Mulgrave, VIC, Australia; heated to 50 °C) was coupled to an electrospray ionization Qtrap 4000 mass spectrometer (ABS SCIEX, Framingham, MA, USA). A binary solvent system with solvent A (60% H20, 20% MeOH and 20% tetrahydrofuran (THF) (*v*/*v*/*v*) with 10 mM ammonium formate) and solvent B (75% THF, 20% MeOH and 5% H_2_O (*v*/*v*/*v*) with 10 mM ammonium formate) was used. A total of 4 lipidomic acquisition methods were used in this study. Two of which were for general lipid profiling, which also monitored lipid classes other than PC and PE and are referred to as general method 1 and 2 (Supplementary Table S7). Two additional methods were developed specifically for flux analysis and focused on PC, PE, LPC, LPE, and sphingomyelin. One flux analysis method included +0 and +4 *m*/*z* for each lipid species detected (+4 method) while the other had +0, +3, +6 and +9 *m*/*z* (+369 method, Supplementary Table S8). The former was used for D4-ethanolamine tracer experiments and the latter for D9-choline and D3-methionine tracer experiments. The run time (20 min) and the detection modes for precursor/product ion pair for each lipid class were identical for all four acquisition methods. These methods had slightly different mass spectrometric voltage settings, as recorded in Supplementary Tables S7 and S8. For general method 1 and the flux analysis methods, the solvent gradient was set to: 90% A, 10% B at time 0; 10 to 100% B from 0 to 13 min; 100% until 16 min; 100 to 10% from 16 to 17 min and 10% B for another 3 min. For general method 2, the solvent gradient was: 90% A, 10% B at time 0; 10 to 20% B from 0 to 0.1 min; 20 to 35% B from 0.1 to 3 min; 35 to 50% B from 3 to 3.5 min; 50 to 62% B from 3.5 to 11 min; 62 to 89% B from 11 to 11.1 min; 89 to 92% B from 11.1 to 14.9 min; 92 to 100% B from 11.9 to 15 min, then 100% until 16.5 min; 100 to 10% from 16.5 to 17 min and 10% B for another 3 min. We adopted this stepped solvent gradient because it allowed a better resolution for lipid peaks of interest. Plasmenyl ether phospholipid species were identified by acid hydrolysis of the vinyl alcohol bond and subsequent removal of the alkenyl chain, as described in our earlier work [43]. AB SCIEX Multiquant software version 2.1.1 (Framingham, MA, USA) was used for peak quantifications and integrations as well as the calculation of lipid concentrations.

### 4.5. Determination of Cell Death

Hepatocyte cell death was determined by propidium iodide staining. In short, 1.2 × 10^6^ hepatocytes were trypsinized and stained with 20 μg/mL propidium iodide (Cayman Chemical, Ann Arbor, MI, USA) PBS solution for 10 min under room temperature, in the dark. The cells were then centrifuged (200× *g*, 10 min) and the supernatant removed, before resuspending in PBS, followed by filtration through a 100 μm mesh. The samples were immediately analyzed with a BD Canto II flow cytometer and the data were acquired and processed with BD FACSDiva software (BD Bioscience ANZ, Sydney, NSW, Australia).

### 4.6. Statistical Analysis

All the experiments were performed with 3–5 biological replicates. For lipidomic experiments, each biological replicate had five technical repeats for each experimental condition, which were averaged. For statistical analysis, a Kruskal–Wallis test was first performed, followed by Mann–Whitney U tests as the *post hoc* analysis, due to the non-parametric and heteroscedastic nature of our datasets. When analyzing the entire lipidomic profile (i.e., Figure 7 and Supplementary Table S6), the *p*-values were corrected for multiple comparison using the Benjamini–Hochberg approach. We have used a corrected *p*-values < 0.05 to indicate significance although the reader will appreciate that our interpretation relied on multiple factors including biology, trends and repeated measures to draw conclusions. All the statistical analyses were carried out with Python v3.6.6 and its scientific and statistical packages (Pandas v0.23.4; Scipy v1.1.0; Numpy v1.14.2; Statsmodels v0.9.0). Python packages Matplotlib v3.0.3 and Seaborn 0.9.0 were used for figure generation.

## 5. Conclusions

In conclusion, our study demonstrates that treating hepatocytes with palmitic and oleic acid 1:2 mixture lowers hepatocyte PC/PE mainly by increasing the cellular PE level and a subtle decrease in the PC level under some experimental conditions. The increase in PE is attributable to enhanced production via the CDP-ethanolamine pathway and less consumption via the PEMT pathway, with the latter pathway also producing less PC. The preferential use of monounsaturated and saturated fatty acyl groups likely drives an enhanced production of PE via the CDP-ethanolamine pathway, as revealed by the tracer experiments. Furthermore, we report here a previously unidentified effect of MFFA on endogenous methionine and possibly *S*-adenosylmethionine, which has an impact on hepatocyte PC level but can be compensated to some extents when there is ample extracellular methionine supply. Our study also provides a comprehensive lipid profile of steatotic hepatocytes and demonstrates that it is possible to manipulate hepatocyte PE level and thus the PC/PE with the addition and removal of ethanolamine. These findings can be linked to changes to phospholipid homeostasis in NAFLD, and can serve as a starting point for future in vivo or clinical studies.

## Data Availability

The data presented in this study are available in this article and supplementary materials.

## Supplementary Materials

The following are available online at https://www.mdpi.com/2218-1989/11/3/188/s1, Figure S1: Differential effects of palmitic acid, oleic acid, and a 1:2 mixture (MFFA) on hepatocyte phospholipid and phosphatidylcholine to phosphatidylethanolamine ratio, Figure S2: The secretion profile of hepatocyte phospholipid and phosphatidylcholine to phosphatidylethanolamine ratio in response to fatty acid treatment, Figure S3: Isotopic labelling profiles for LPE LPC and SM in control and MFFA treated hepatocytes, Table S1: Phospholipid secretion profile in response to MFFA treatment, Table S2: Phospholipid profile of D4-ethanolamine labelled control and steatotic hepatocytes, Table S3: The time effects of MFFA on hepatocyte phospholipidome, Table S4: Phospholipid profile of D9-choline labelled control and steatotic hepatocytes, Table S5: Phospholipid profile of D3-methionine labelled control and steatotic hepatocytes, Table S6: The impacts of MFFA and altered media nutrients on hepatocyte lipidome, Table S7: Instrumental settings for general lipidomics method 1 and 2, Table S8: Instrumental settings for tracer phospholipidomics methods.

## Abbreviations

CDP	cytidine diphosphate
FFA	free fatty acid
LPC	lysophosphatidylcholine
LPE	lysophosphatidylethanolamine
MFFA	mixed free fatty acid
NAFLD	non-alcoholic fatty liver disease
PC	phosphatidylcholine
PC/PE	phosphatidylcholine to phosphatidylethanolamine ratio
PE	phosphatidylethanolamine
PEMT	phosphatidylethanolamine *N*-methyl transferase
SM	sphingomyelin
