# Supplementary material for: Stable Isotopic Tracer Phospholipidomics Reveals Contributions of Key Phospholipid Biosynthetic Pathways to Low Hepatocyte Phosphatidylcholine to Phosphatidylethanolamine Ratio Induced by Free Fatty Acids
Source: Metabolites. 2021 Mar 22;11(3):188. doi: 10.3390/metabo11030188 (PMC8004269; doi:10.3390/metabo11030188)
Supplement: Supplementary file 1 [file metabolites-11-00188-s001.zip › Peng et al_Supplementary Information_revision.docx]

**Stable Isotopic Tracer Phospholipidomics Reveals Contributions of Key Phospholipid Biosynthetic Pathways to Low Hepatocyte Phosphatidylcholine to Phosphatidylethanolamine Ratio Induced by Free Fatty Acids**

Kang-Yu Peng (彭康祐)1,2, Chris K Barlow1,3, Helene Kammoun4, Natalie A Mellett1, Jacquelyn M Weir1, Andrew J Murphy4, Mark A Febbraio5 and Peter J Meikle1, 6*

^1^ Metabolomics Laboratory, Baker Heart Institute, Melbourne, Victoria, Australia

^2^ Department of Biochemistry and Molecular Biology, University of Melbourne, Parkville, Victoria, Australia

^3^ Proteomics and Metabolomics Facility and the Department of Biochemistry and Molecular Biology, Biomedicine Discovery Institute, Monash University, Clayton, VIC 3800, Australia

^4^ Hematopoiesis & Leukocyte Biology Laboratory, Baker Heart Institute, Melbourne, Victoria, Australia

^5^ Cellular & Molecular Metabolism Laboratory, Drug Discovery Biology, Monash Institute of Pharmaceutical Sciences, Monash University, Melbourne, Victoria, Australia

^6^ Baker Department of Cardiometabolic Health, University of Melbourne, Parkville, Victoria, Australia

* Correspondence: peter.meikle@baker.edu.au; Tel.: 613 8532 1770

ORCID ID:

K Peng: 0000-0003-4024-4088

P Meikle: 0000-0002-2593-4665

**Supplementary Information**

**Supplementary Materials**

For materials used in this study, bicinchoninic acid (BCA) assay kit was purchased from Pierce Biochemical (Rockford, IL, USA). Butanol was purchased from Merck & Co. (Kenilworth, NJ, USA). Collagenase A was from Roche Diagnostics (Rotkreuz, Switzerland). D3-L-methionine (methyl-D3), D4-ethanolamine (1,1,2,2-D4) and D9-choline chloride (trimethyl-D9) were purchased from Cambridge Isotopes (Tewksbury, MA, USA). DMEM without L-glutamine, L-methionine, calcium, D-pentothenic acid, choline chloride, inositol and sodium pyruvate (Cat. D9809; called special DMEM in the later context) was from US Biological Life Sciences (Salem, MA, USA). M199 media was purchased from Life Technologies (Carlsbad, CA, USA). Propidium iodide solution was from Cayman Chemical (Ann Arbor, MI, USA). Ultroser^TM^ G serum substitute was from Pall Corporation (New York, USA). All the other chemicals, reagents and solvents were obtained from Sigma-Aldrich (St. Louis, MO, USA). Solvents for lipid extraction and LC-MS lipidomics analysis were of LC-MS grade. For lipid internal standards, cholesterol ester 18:0 (d6) was from C/D/N Isotopes (Pointe-Claire, Quebec, Canada). Diacylglycerol 15:0/15:0 and triacylglycerol 17:0/17:0/17:0 were from Sigma-Aldrich. Dihexosylceramide 16:0 (d3), monohexosylceramide 16:0 (d3) and trihexosylceramide 17:0 were purchased from Matreya LCC Inc. (State College, PA, USA). All the other lipid internal standards were from Avanti Polar Lipids Inc. (Alabaster, AL, USA).

**Supplementary Experimental Procedures**

*Rat Hepatocytes Isolation*

Briefly, male Sprague Dawley rats between 6~10 weeks of age were anaesthetised with pentobarbitone, followed by portal vein cannulation. Livers were perfused with 400 mL of HEPES buffer (10 mM HEPES, 137 mM NaCl, 3.5 mM KCl, 0.7 mM Na_2_HPO_4_; pH 7.0-7.4; 37°C) followed by 300~400 mL of HEPES buffer containing 0.18 mg/mL collagenase A and 0.22 mg/mL CaCl_2_, using a peristaltic pump (Harvard Apparatus, MA, USA) with a flow rate set at 44 mL/min. The perfused liver was then excised, each lobe was incised, and the hepatocytes were released and suspended into warm HEPES buffer. The cells were then filtered with a 250 μm mesh, washed with HEPES buffer and filtered again through a 100 μm mesh. Trypan blue staining indicated that the cell viability was 70% or higher. Cells were seeded at a density of 1.2 x 10^6^ cells per well in 6-well culture plates in M199 media containing 5000 U penicillin/streptomycin, 0.1% BSA, 2% Ultroser^TM^ G serum substitute, 100 nM dexamethasone, 100 nM thyrodoxin and 1 nM insulin. After 3-5 hours of plating, the media was replaced with M199 containing 5000 U penicillin/streptomycin and 100 nM dexamethasone and incubated overnight (37°C) before the hepatocytes were ready for experiments.

*Preparation of Fatty acid-BCA Conjugation*

100 mM of palmitic acid and oleic acid stock solutions were prepared in 0.1 N NaOH by heating to 70°C, to ensure complete solubilisation, prior to filtration with 0.22 μm syringe filters. 100 mM palmitic acid, oleic acid, or the 1:2 mixture of the two prepared with the above FFA stock solutions was slowly dripped into 13.6% 0.22 μm-syringe-filtered, delipidated bovine serum albumin (BSA) solution under constant shaking. To allow full complex formation, the solution was then incubated in a 60°C water bath for 1 hour, which gave us BSA-FFA complex solution containing 12 mM FFA and 12 % BSA. To prepare BSA-FFA complex solution with lower concentrations, we simply diluted the BSA-FFA solution with a 12% BSA solution. Before cell treatment, the FFA-BSA complex solution with a free fatty acid concentration 6 times of the desired final concentration was mixed with culture media in 1:5 ratio. The final concentrations of media free fatty acid and BSA were 0.67-2.00 mM (depending on the experiment) and 2%, respectively. Media for the control group was similarly prepared by mixing pure culture media with 12% BSA solution containing the same amount of 0.1 N NaOH, but without FFA.

**Supplementary Figures**

**Supplementary Figure 1. Differential effects of palmitic acid, oleic acid, and a 1:2 mixture (MFFA) on hepatocyte phospholipid and phosphatidylcholine to phosphatidylethanolamine ratio**

Isolated rat hepatocytes were treated with palmitic acid (0.67 or 2 mM), oleic acid (1.33 or 2 mM) or 1:2 mixture of the aforementioned fatty acids (MFFA, 2 mM) for 24 hours. Phosphatidylcholine and phosphatidylethanolamine contents (nmol/mg protein) in cellular extracts were determined by LC-MS lipidomic analysis, and the phosphatidylcholine to phosphatidylethanolamine ratio (PC/PE) was calculated accordingly. Control group (Ctrl) are hepatocytes cultured under the same condition for 24 hours, but without the addition of fatty acid. Data shown as mean ± sem; a: *p<0.05* vs Ctrl group, as determined by Mann-Whitney U test; n=3 for Ctrl, MFFA (2 mM), oleic acid (2 mM) and palmitic acid (2 mM); n=2 for oleic acid (1.33 mM) and palmitic acid (0.67 mM).

**Supplementary Figure 2. The secretion profile of hepatocyte phospholipid and phosphatidylcholine to phosphatidylethanolamine ratio in response to fatty acid treatment**

Cellular culture media were collected from experiments described in Figure 2 at the chosen time points and media phosphatidylcholine and phosphatidylethanolamine concentrations (in nmol/ml media) were extracted and determined by LC-MS (A and B). phosphatidylcholine to phosphatidylethanolamine ratio (PC/PE) was calculated accordingly (C). Data shown as mean ± sem; a: *p<0.05* vs 0.0 mM group as determined by Mann-Whitney U test (n=4).

**Supplementary Figure 3. Isotopic labelling profiles for LPE LPC and SM in control and MFFA treated hepatocytes.**

Isolated rat hepatocytes were treated without (Ctrl) or with 2 mM of MFFA in special DMEM media of which a nutrient has been replaced with its deuterated version (50 μM D4-ethanolamine, 30 μM D9-choline or 200 μM D3-methionine), serving as a flux tracer for phospholipid biosynthetic pathways, for 6 hours. The cells were then harvested and underwent lipid extraction, followed by lipidomic analysis. (A) the concentrations of unlabelled (unlab.) and D4-labelled (D4) LPE in the D4-ethanolamine labelling experiments (Data shown as mean ± sem; pink: D4-labelled LPE; n=4). S. Figure 4B and 4C show the concentrations of the unlabelled (unlab.) and D3, D6 and D9-labelled (D3, D6 and D9) LPC and SM in D9-choline and D3-methionine labelling experiments, respectively. (Data shown as mean ± sem; discrete Y-axis; blue: D3-labelled lipid; orange: D6-labelled lipid; green: D9-labelled lipid; a: *p<0.05* vs corresponding unlabelled/labelled LPC/SM in Ctrl group; Mann-Whitney U test; n=3 for D9-choline and D3-methionine experiments).
